# Correction to ‘Individual-based model of juvenile eel movement parametrized with computational fluid dynamics derived flow fields informs improved fish pass design’

**DOI:** 10.1098/rsos.200158

**Published:** 2020-02-12

**Authors:** Thomas E. Padgett, Robert E. Thomas, Duncan J. Borman, David C. Mould

*R. Soc. open sci.* **7**, 191505. (Published Online 15 January 2020) (doi:10.1098/rsos.191505)

This correction refers to errors in the labelling of the colour scale in figure 8. ‘Velocity u (m s^−1^)’ was erroneously changed to ‘Velocity μ (m s^−1^)’. This has now been corrected.

