# Supplementary material for: Individual-based model of juvenile eel movement parametrized with computational fluid dynamics-derived flow fields informs improved fish pass design
Source: R Soc Open Sci. 2020 Jan 15;7(1):191505. doi: 10.1098/rsos.191505 (PMC7029934; doi:10.1098/rsos.191505)
Supplement: Padgett et al_Supplementary_Captions_Final.docx [file rsos191505supp4.docx]

Supplementary Figure 1: Classified time-averaged velocity fields extracted in a plane 3 mm above the floor of an eel tile installed at 8°, 11°, 14°, 17°, and 20° for discharges per unit width of 1.67, 3.33 and 5.0 × 10^-3^ m^2^s^−1^. Classified using the median burst speed of elver of length 0.05 m. By area, over 99.9% of the domain is passable at an installation angle of 8° and a discharge per unit width of 1.67 × 10^-3^ m^2^s^−1^, while only 40.2% of the domain is passable at an installation angle of 20° and a discharge per unit width of 5.0 × 10^-3^ m^2^s^−1^. Although 57% of the domain is passable at an installation angle of 20° and a discharge per unit width of 3.33 × 10^-3^ m^2^s^−1^, there is no continuous passable path from the downstream end of the pass to the upstream end of the pass and thus it is not possible for an elver to ascend. This is also true for passes inclined at 17° and 20° at a discharge per unit width of 5.0 × 10^-3^ m^2^s^−1^. Installation angle is shown to the left, discharge per unit width to the top. White denotes “passable”, red denotes “impassable”, black denotes “studs”, and grey denotes “boundary”. Note that boundaries are one cell thick and so appear as lines in the figure.

Supplementary Figure 2: Classified time-averaged velocity fields extracted in a plane 3 mm above the floor of an eel tile installed at 8°, 11°, 14°, 17°, and 20° for discharges per unit width of 1.67, 3.33 and 5.0 × 10^-3^ m^2^s^−1^. Classified using the median burst speed of elver of length 0.07 m. By area, 100% of the domain is passable at an installation angle of 8° and a discharge per unit width of 1.67 × 10^-3^ m^2^s^−1^, while 74.8% of the domain is passable at an installation angle of 20° and a discharge per unit width of 5.0 × 10^-3^ m^2^s^−1^. Consideration of whether an unbroken passable path is present indicates that all of the tested installation angle and discharge per unit width combinations are passable by elvers of this length or longer. Installation angle is shown to the left, discharge per unit width to the top. White denotes “passable”, red denotes “impassable”, black denotes “studs”, and grey denotes “boundary”. Note that boundaries are one cell thick and so appear as lines in the figure.

Supplementary Figure 3: Classified time-averaged velocity fields extracted in a plane 3 mm above the floor of an eel tile installed at 8°, 11°, 14°, 17°, and 20° for discharges per unit width of 1.67, 3.33 and 5.0 × 10^-3^ m^2^s^−1^. Classified using the median burst speed of elver of length 0.09 m. By area, 100% of the domain is passable at an installation angle of 8° and a discharge per unit width of 1.67 × 10^-3^ m^2^s^−1^, while 96% of the domain is passable at an installation angle of 20° and a discharge per unit width of 5.0 × 10^-3^ m^2^s^−1^. Consideration of whether an unbroken passable path is present indicates that all of the tested installation angle and discharge per unit width combinations are passable by elvers of this length or longer. Installation angle is shown to the left, discharge per unit width to the top. White denotes “passable”, red denotes “impassable”, black denotes “studs”, and grey denotes “boundary”. Note that boundaries are one cell thick and so appear as lines in the figure.
